# Circulating EGFL7 distinguishes between IUGR and PE: an observational case–control study

**DOI:** 10.1038/s41598-021-97482-2

**Published:** 2021-09-09

**Authors:** Micol Massimiani, Silvia Salvi, Grazia M. Tiralongo, Sascia Moresi, Heidi Stuhlmann, Herbert Valensise, Antonio Lanzone, Luisa Campagnolo

**Affiliations:** 1 Saint Camillus International, University of Health Sciences, Via di Sant’Alessandro 8, 00131 Rome, Italy; 2 Department of Biomedicine and Prevention, University of Rome Tor Vergata, Via Montpellier 1, 00133 Rome, Italy; 3 Dipartimento Scienze della Salute della Donna, del Bambino e di Sanità Pubblica, UOC di Patologia Ostetrica, Fondazione Policlinico Universitario “A Gemelli’’ IRCCS, Largo Agostino Gemelli 8, 00168 Rome, Italy; 4 Department of Obstetrics and Gynaecology, Casilino General Hospital, Via Casilina 1049, 00169 Rome, Italy; 5 Department of Cell and Developmental Biology, Weill Cornell Medical College, 1300 York Avenue, Box 60, New York, NY 10065 USA; 6 Obstetrics and Gynaecology Unit, Department of Surgical Sciences, University of Rome Tor Vergata, Via Montpellier 1, 00133 Rome, Italy; 7 Università Cattolica del Sacro Cuore, Largo Francesco Vito 1, 00168 Rome, Italy

**Keywords:** Developmental biology, Biomarkers, Diseases, Pathogenesis, Signs and symptoms

## Abstract

Isolated intrauterine growth restriction (IUGR) and preeclampsia (PE) share common placental pathogenesis. Differently from IUGR, PE is a systemic disorder which may also affect liver and brain. Early diagnosis of these conditions may optimize maternal and fetal management. Aim of this study was to assess whether Epidermal Growth Factor-Like domain 7 (EGFL7) dosage in maternal blood discriminates between isolated IUGR and PE. A total of 116 women were enrolled in this case–control study: 12 non-pregnant women, 34 healthy pregnant women, 34 women presenting with isolated IUGR and 36 presenting with PE. Levels of circulating EGFL7 and other known pro- and anti-angiogenic factors were measured by ELISA at different gestational ages (GA). Between 22–25 weeks of gestation, EGFL7 levels in early-onset PE (e-PE) plasma samples were significantly higher than those measured in controls or isolated IUGR samples (69.86 ± 6.17 vs. 19.8 ± 2.5 or 18.8 ± 2.8 µg/ml, respectively). Between 26–34 weeks, EGFL7 levels remained significantly higher in e-PE compared to IUGR. At term, circulating and placental EGFL7 levels were comparable between IUGR and late-onset PE (l-PE). In contrast, circulating levels of PlGF were decreased in both IUGR- and PE- complicated pregnancies, while levels of both sFLT-1 and sENDOGLIN were increased in both conditions. In conclusion, EGFL7 significantly discriminates between isolated IUGR and PE.

## Introduction

The kinetics of placental and fetal growth are closely interrelated and are both important features predicting prenatal and postnatal health of the newborn, and cardiovascular adaptations in childhood^1,2^.

Intrauterine growth restriction (IUGR) is defined as the failure of the fetus to achieve its genetically determined growth potential^3^. Different underlying causes may explain IUGR, but the majority of cases not associated with congenital malformations, genetic anomalies, or infectious etiology are thought to arise from compromised utero-placenta circulation. Main features of the placental tissues in IUGR are a reduction in the volume and surface area, and inadequate vascularization of the intermediate and terminal villi, that mediate maternal–fetal exchange^4–6^. Although IUGR and preeclampsia (PE) share common features such as ischemic placental disease, diffuse maternal endothelial damage and inflammation, the main features of preeclamptic syndrome, are lacking in isolated IUGR. However, severe and early-onset isolated IUGR remain to be challenging fetal complications, since they predispose to fetal death, neonatal death, neonatal morbidity and neurodisability.

Recent evidence suggests that different angiogenic biomarkers are involved in the pathogenesis of PE. Thus, their assessment could be clinically useful in the prediction of the disease onset, severity and timing of delivery. Epidermal Growth Factor Like Domain 7 (EGFL7) is a largely endothelial-restricted secreted factor that is critical for embryonic vascular development. In humans the Egfl7 gene is located on chromosome 9, consists of 11 exons^7,8^, and contains miR126, located in intron 7^9–11^. It encodes a 29 kDa protein, encompassing a putative amino-terminal signal peptide domain, an EMI-like domain, and 2 centrally located EGF-like domains^7,8^. Egfl7 was originally identified as an endothelial restricted gene, showing high expression levels in proliferating endothelial cells during embryogenesis and physiologic and pathologic angiogenesis^7,8,12–14^. A role for EGFL7 has been demonstrated in vascular development, where it promotes endothelial cell proliferation, adhesion, migration, sprouting, and invasion^12–16^. More recently, other sources of EGFL7 have been identified, such as primordial germ cells, adult neural stem cells, cancer cells of various human tumors and trophoblast cells^17–21^. We have previously demonstrated that EGFL7 is expressed in human placental villi by endothelial cells, as well as by cytotrophoblast and syncytiotrophoblast cells, and that it regulates trophoblast migration and invasion^21,22^, suggesting a potential involvement in placental development. EGFL7 functions by modulating the Notch and Epidermal Growth Factor Receptor (EGFR) pathways and binds integrin α_v_β_3_^7,12,21–23^. Moreover, we showed that circulating levels of EGFL7 increase during normal pregnancy and declines toward term^24^. Interestingly, in pregnant women affected by PE placental levels of EGFL7 are down-regulated^21,25^, whereas its levels are significantly increased in the maternal circulation at term (i.e., 35–40 weeks of gestation)^24^. Of note, by principal component analysis (PCA), we demonstrated that EGFL7 distributes as a separate factor with respect to the three well-known angiogenic factors Placental Growth Factor (PlGF), soluble fms-like tyrosine kinase 1 (sFLT-1) and soluble Endoglin (sENDOGLIN), indicating that it may represent an additional independent diagnostic marker of PE^24^.

The aim of this study was to investigate whether EGFL7 may be marker that can discriminate between isolated IUGR and PE. To this end, circulating levels of EGFL7 were compared between pregnancies complicated by isolated IUGR and uncomplicated and PE-affected pregnancies. Levels of sFLT-1, sENDOGLIN and PlGF were also investigated.

## Methods

### Patients

The proposed study is an observational case–control study. Women with a diagnosis of IUGR and PE were included as cases, while non-pregnant and healthy pregnant women with uncomplicated pregnancies and normally grown fetuses were included as controls. A total of 116 women were enrolled in this case–control study, of which 12 were non-pregnant, 34 were healthy pregnant, 34 presenting with isolated IUGR and 36 presenting PE (29 with early-onset PE, e-PE, and 7 with late-onset PE, l-PE). Only singleton pregnancies were enrolled in the study.

Pregnancies were considered complicated by IUGR in case of an estimated fetal weight < 10th percentile together with pulsatility index in the umbilical artery > 95° percentile and/or cerebro-placental ratio < 5° percentile, according to the definitions from Gordjin et al.^26^. Cases of IUGR caused by genetic factor, infection, or maternal diseases were excluded from the IUGR group.

Women were diagnosed as PE in the presence of a new-onset high blood pressure (> 140/90 mmHg in more than two occasions at a distance of four hours) with proteinuria (> 0.3 g/24-h in 24-h urine collection). In the absence of proteinuria, PE was diagnosed in the presence of hypertension associated to thrombocytopenia (platelet count < 100 000/μL), impaired liver function (raised blood levels of liver aminotransferases to twice the normal concentration), new development of renal insufficiency (elevated serum/creatinine > 1.1 mg/dL), pulmonary edema, new-onset cerebral or visual disturbances or signs of placental insufficiency and fetal growth restriction^27^. According to the gestational age (GA) at diagnosis, women affected by PE were divided in two groups: e-PE (n = 29) if the diagnosis was performed early than 34 weeks and l-PE (n = 7) if the diagnosis was performed later than 34 weeks. Among e-PE, 28 women have also a diagnosis of IUGR (28/29: 96.5%).

Women with uncomplicated pregnancies were included as controls and underwent to serial assessment from conception to delivery. In particular, in healthy pregnant women, the analysis of EGFL7 was performed throughout the entire gestation, within intervals of 3–4 gestational weeks, between 8 and 40 weeks. For each interval, at least five samples were analyzed.

Maternal age, body mass index (BMI) and ethnicity, gravidity and parity, highest systolic and diastolic blood pressure, maximum proteinuria value for all women were registered. Data on pregnancy outcome were also collected from the electronic hospital obstetric and neonatal records. The perinatal outcome variables investigated were GA at delivery, birthweight (g) and birthweight percentile, calculated according to Yudkin et al.^28^, and Apgar Score at 1st and 5th minute. GA was established by ultrasound dating by crown rump length prior to 14 weeks of gestation; patients with uncertain GA were not included.

Plasma and placental samples were obtained from the Fondazione Policlinico Agostino Gemelli, IRCCS, and Policlinico Casilino, Rome, Italy. The study respected the principles expressed in the Declaration of Helsinki and was approved by the Bioethical Committee of the Catholic University of Sacred Heart of Rome, Italy (approval number P575(A1420)/CE/2008) and the Bioethical Committee of the ASL Roma2, Italy (approval number 0069765 of April 21, 2017). Informed written consent was obtained from each patient.

### Biological samples processing

Plasma and placental samples were collected under the protocol approved by the Bioethical Committee of the Catholic University of Sacred Heart of Rome and the Bioethical Committee of the ASL Roma2, Italy.

Briefly, peripheral venous blood was collected into ethylenediaminetetraacetic acid tubes and processed within one hour. Blood samples were centrifuged at 1500 × g for 10 min at 4 °C. Plasma was aliquoted and stored at − 80 °C until analysis.

Placentas were collected during elective caesarean section. Briefly, placental samples were obtained by multiple biopsies in the parasagittal plane with respect to the umbilical cord insertion. We obtained a total of 22 placentas, 11 placentas from healthy controls, 8 from patients with IUGR and 3 from patients affected by l-PE. All samples were immediately frozen in liquid nitrogen and stored at − 80 °C until analysis.

### Enzyme-linked immunosorbent assays

Enzyme-linked immunosorbent assays (ELISAs) were performed using commercial kits. Human EGFL7 kits were purchased from Cusabio Biotech (College Park, Maryland, USA). Human sFLT-1, free PlGF, and sENDOGLIN kits were purchased from R&D Systems (Minneapolis, Minnesota, USA). Assays were performed according to the manufacturer’s instructions in the Department of Biomedicine and Prevention, University of Rome Tor Vergata, Italy. Data on spike/recovery, inter- and intra- assay precision were provided by the manufacturer. In details, intra-assay precision was indicated as coefficient of variability (CV) % and calculated as < 8% by testing three samples of known concentration twenty times on one plate. Inter-assay precision was reported as CV% < 10% and it was obtained by testing three samples of known concentration in twenty assays. To confirm these data, we tested five different plasma samples in at least three different assays and obtained comparable results. In each ELISA, all samples and standards have been tested in duplicate; duplicates always gave similar values of optical density (OD).

### Real-time PCR

RNA from placental samples was prepared using the TRIZOL Reagent (Roche Diagnostics GmbH, Mannheim, Germany), according to the manufacturer’s protocol. RNA quality was assessed by evaluating the presence of ribosomal RNA bands on agarose gels. RNA was reverse transcribed using random primers and the QuantiTect Reverse Transcription Kit (Qiagen, Hilden, Germany) following the manufacturer’s specifications. Gene expression was measured using iTaq Universal SYBR Green Supermix (Biorad Laboratories, Hercules, CA). Real-time PCR was performed using LightCycler 96 Real Time PCR System (Roche Diagnostics GmbH). Differences among gene expression were quantified using the ΔΔCt method with normalization to 18S ribosomal RNA gene. Specific intron-spanning primers for EGFL7 and 18S were designed using Primer Express software (Applied Biosystems in Life Technologies, Monza, Italy). Primer sequences are listed below:

- EGFL7 (forward) 5’-TCGTGCAGCGTGTGTACCAG-3’
- EGFL7 (reverse) 5’-GCGGTAGGCGGTCCTATAGATG-3’
- 18S (forward) 5’-GAGGCCCTGTAATTGGAATGAG-3’
- 18S (reverse) 5’-GCAGCAACTTTAATATACGCTATTGG-3’

### Statistical analysis

Continuous data were presented as median and interquartile range IQR or as mean and standard deviation when normally distributed. The Kolmogorov–Smirnov test was used to assess the normality of the distribution of the data. Normally distributed data were compared using the two-sample *t*-test between two samples or ANOVA test among more than two groups. A nonparametric analysis (Mann–Whitney U test) was used to compare not normally distributed data.

Asterisks indicate the level of statistical significance (*p = 0.05; **p < 0.05; ***p < 0.001; ****p < 0.0001). SPSS v20 was used for statistical analysis of the clinical findings, SigmaPlot 12.0 and GraphPad Prism 7 were used for statistical analysis of the ELISA experiments.

## Results

### Maternal and perinatal findings

Maternal and perinatal outcome findings are described in Tables 1 and 2. There were no statistically significant differences among groups regarding maternal age, gravidity and parity as determined by one-way ANOVA. Maternal BMI was statistically different among groups with higher values detectable in women with l-PE, whereas lower values characterized women with e-PE (ANOVA test among the groups *p* < 0.001). Higher blood pressure and proteinuria were found in pregnancies with PE (ANOVA test among the groups *p* < 0.001). Statistically significant difference in perinatal outcomes in terms of GA at delivery, neonatal weight and neonatal weight centile was found, with pregnancies complicated by IUGR and e-PE having significantly decreased values (ANOVA test among the groups *p* < 0.001). A significant lower GA at delivery and neonatal birth weight were demonstrated in e-PE when compared to IUGR (*p* < 0.001), whereas no statistically significant difference could be observed in neonatal birth weight percentile and neonatal Apgar at 1st and 5th minute.

**Table 1:** Maternal findings of the studied pregnancies.

	Controls N° 34	IUGR N° 34	*p*-value^§^	e-PE N° 29	*p*-value^§§^	*p*-value^$^	l-PE N° 7	*p*-value^§§§^	*p*-value^£^
Maternal age (years)	32.7 ± 4.9	33.4 ± 5.7	0.568	36.2 ± 5.1	**0.008**	**0.048**	34.0 ± 4.2	0.508	0.059
Body Mass Index (BMI) (kg/m^2^)	23.8 ± 3.2	25.9 ± 4.2	**0.031**	23.3 ± 3.5	0.540	**0.012**	30.9 ± 3.7	** < 0.0001**	** < 0.0001**
Ethnicity	Caucasian 100%	Caucasian 100%	–	Caucasian 100%	–	–	Caucasian 100%	–	–
Highest systolic blood pressure (mmHg)	108.7 ± 15.9	118.8 ± 16.6	**0.012**	147.8 ± 20.0	** < 0.0001**	** < 0.0001**	151.4 ± 10.7	** < 0.0001**	** < 0.0001**
Highest diastolic blood pressure (mmHg)	69.3 ± 8.7	67.9 ± 13.2	0.604	94.6 ± 12.4	** < 0.0001**	** < 0.0001**	100.7 ± 10.2	** < 0.0001**	** < 0.0001**
Maximum proteinuria value (g/L)	0.1 ± 0.2	0.1 ± 0.2	0.173	4.2 ± 3.4	** < 0.0001**	** < 0.0001**	1.7 ± 0.7	** < 0.0001**	** < 0.0001**
Gravidity	1.8 ± 1.1	1.9 ± 1.2	0.668	1.8 ± 1.5	0.866	0.603	1.4 ± 0.8	0.343	0.755
Parity	0.3 ± 0.7	0.5 ± 0.8	0.214	0.3 ± 0.7	0.967	0.255	0.0 ± 0.0	0.253	0.256

^§^Two-independent sample *t*-test (IUGR vs. Controls).

^§§^Two-independent sample *t*-test (e-PE vs. Controls).

^$^Two-independent sample *t*-test (IUGR vs. e-PE).

^§§§^Two-independent sample *t*-test (l-PE vs. Controls).

^£^Anova Test among the four groups (Controls vs. IUGR vs. e-PE vs. l-PE).

*IUGR* Intrauterine Growth Restriction; *PE* Preeclampsia; *e-PE* Early-onset PE; *l-PE* Late-onset PE.

**Table 2:** Perinatal outcome of the studied pregnancies.

	Controls N° 34	IUGR N° 34	*p*-value^§^	e-PE N° 29	*p*-value^§§^	*p*-value^$^	l-PE N° 7	*p*-value^§§§^	*p*-value^£^
Gestational age at delivery (weeks)	39.4 ± 1.3	34.9 ± 3.5	** < 0.0001**	32.1 ± 2.1	** < 0.0001**	** < 0.0001**	37.3 ± 1.1	**0.001**	** < 0.0001**
Birthweight (grams)	3351.8 ± 406.1	1894.4 ± 632.5	** < 0.0001**	1271.0 ± 379.5	** < 0.0001**	** < 0.0001**	2562.8 ± 556.4	** < 0.0001**	** < 0.0001**
Birthweight centile (Yudkin)	44.2 ± 26.8	7.6 ± 11.7	** < 0.0001**	3.6 ± 7.2	** < 0.0001**	0.137	20.9 ± 24.1	**0.040**	** < 0.0001**
Apgar 1st minute	8.7 ± 0.8	8.1 ± 1.8	0.094	7.4 ± 1.4	** < 0.0001**	0.069	8.0 ± 1.8	0.733	**0.005**
Apgar 5th minute	9.7 ± 0.5	9.2 ± 1.0	**0.014**	8.8 ± 0.8	** < 0.0001**	0.102	9.4 ± 0.8	0.215	** < 0.0001**

^§^Two-independent sample *t*-test (IUGR vs. Controls).

^§§^Two-independent sample *t*-test (e-PE vs. Controls).

^$^Two-independent sample *t*-test (IUGR vs. e-PE).

^§§§^Two-independent sample *t*-test (l-PE vs. Controls).

^£^Anova Test among the four groups (Controls vs. IUGR vs. e-PE vs. l-PE).

*IUGR* Intrauterine Growth Restriction; *PE* Preeclampsia; *e-PE* Early-onset PE; *l-PE* Late-onset PE.

### Levels of circulating EGFL7

**Figure 1:**
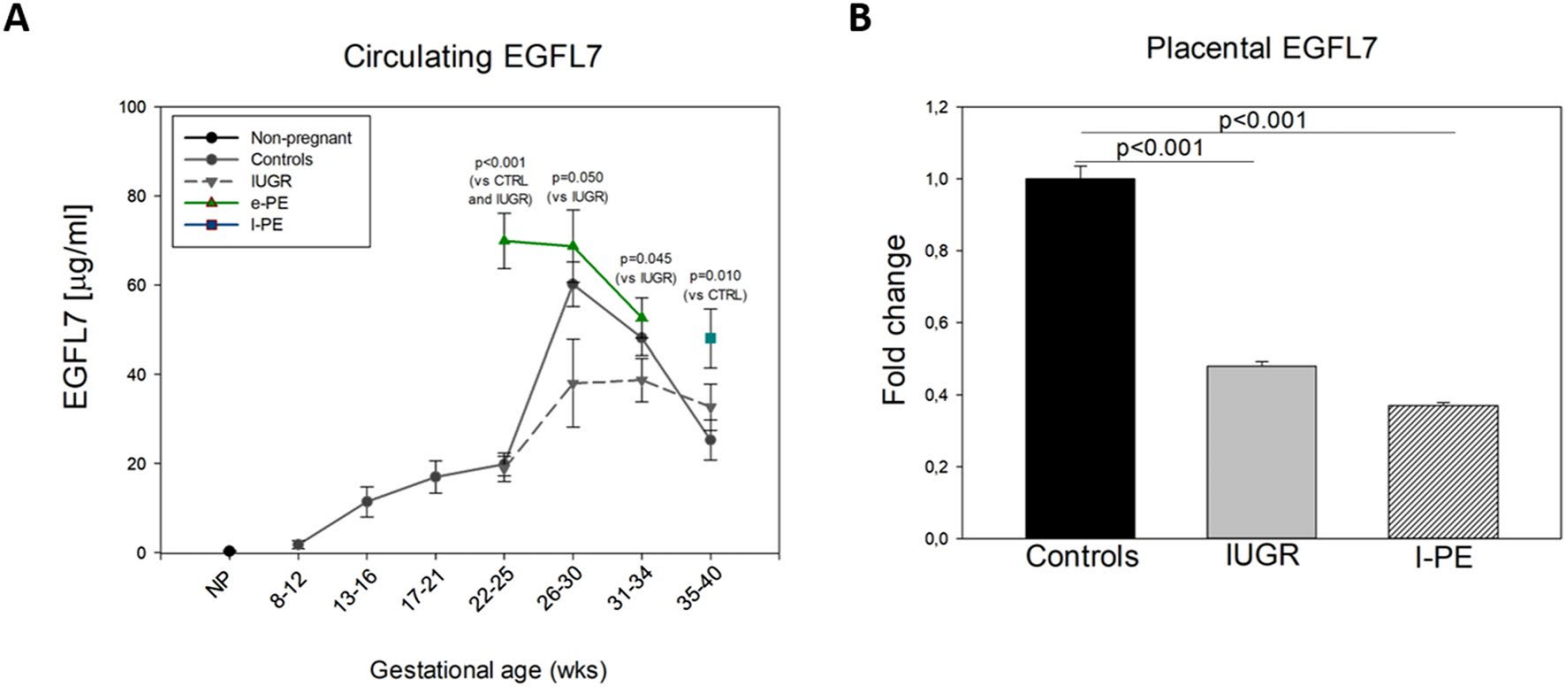
EGFL7 levels in non-pregnant women, healthy pregnant women, and pregnant women affected by IUGR, Early PE and Late PE. (**A**) Mean (± SE) levels of Epidermal Growth Factor-like Domain 7 (EGFL7) in plasma of non-pregnant women (NP), healthy pregnant women (Controls), IUGR-, Early PE- (e-PE) and Late PE- (l-PE) affected pregnant women throughout pregnancy as measured by ELISA. Data were compared using the two-sample *t*-test between two samples (*p* < 0.001 for e-PE vs. CTRL and IUGR at GA 22–25; *p* = 0.05 for e-PE vs. CTRL and IUGR at GA 26–30; *p* = 0.045 for e-PE vs. IUGR at GA 31–34; *p* = 0.01 for l-PE vs. CTRL) or ANOVA test among the three studied groups (*p* < 0.001 at GA 22–25 and *p* = 0.036 at GA 35–40). Statistically significant difference within gestational interval is reported. SigmaPlot 12.0 and GraphPad Prism 7 were used for statistical analysis. (**B**) Real-time PCR analysis for EGFL7 gene expression levels in placental samples obtained from Controls, IUGR- and l-PE- affected pregnant women at GA 35–40. Data were represented as mean (± SE). ANOVA test with post-hoc Bonferroni correction was used for statistical analysis (SigmaPlot 12.0). Differences were considered significant at *p* < 0.05.

We performed a cross-sectional analysis of plasma samples obtained within GA intervals of three to four weeks (Fig. 1A). Consistent with our previous studies, EGFL7 concentrations were undetectable in non-pregnant women and were first measurable between 8 and 12 weeks of gestation in healthy controls, increasing up to 26–30 gestational weeks and then declining until delivery^24^. In samples obtained from e-PE after the onset of clinical signs, circulating levels of EGFL7 were significantly increased at 22–25 weeks of gestation compared to both isolated IUGR and controls (69.86 ± 6.17 µg/ml, 18.8 ± 2.8 µg/ml and 19.8 ± 2.5 µg/ml, respectively; Fig. 1A). At later stages (26–30 and 31–34), such increase was maintained but was not statically significant when compared to controls. In women with isolated IUGR-complicated pregnancies, EGFL7 levels were lower compared to uncomplicated pregnancies at all gestational intervals up to 34 weeks of gestation, although statistical significance was not met. However, EGFL7 levels in isolated IUGR were significantly lower when compared to levels measured in women affected by e-PE (Fig. 1A). Of note, differences between isolated IUGR and e-PE were highly significant at 22–25 weeks of gestation (*p* < 0.001). At term (i.e., GA 35–40) the difference between circulating levels of EGFL7 in isolated IUGR and l-PE was not significant (Fig. 1A), while, as previously reported^12^, a statistically significant difference was observed between l-PE and controls (*p* = 0.010).

### Levels of placental EGFL7

Expression of EGFL7 was measured by real-time PCR in a subset of placental samples collected a term from healthy controls, IUGR- and l-PE- complicated pregnancies (i.e., GA 35–40; Fig. 1B). When compared to normal placentas, EGFL7 mRNA expression was significantly decreased in both isolated IUGR (*p* < 0.001) and l-PE samples (*p* < 0.001; Fig. 1B). Similarly to what observed in plasma samples collected at term, the difference between levels of EGFL7 in isolated IUGR and l-PE placentas was not significant (Fig. 1B).

### Levels of circulating PlGF, sFLT-1 and sENDOGLIN

**Figure 2:**
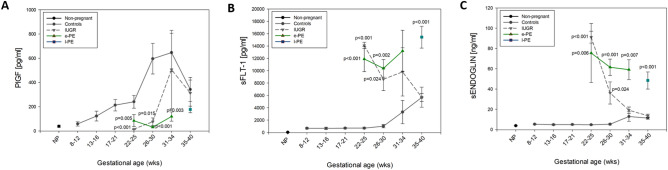
PlGF, sFLT-1 and sENDOGLIN levels in non-pregnant, healthy pregnant women, IUGR-, Early PE- and Late PE- affected pregnant women along gestation. Mean (± SE) levels of Placental Growth Factor (PlGF, (**A**), soluble fms-like tyrosine kinase 1 (sFLT-1, (**B**) and soluble Endoglin (sENDOGLIN, (**C**) in plasma of non-pregnant women (NP), healthy pregnant women (Controls), IUGR-, Early PE- (e-PE) and Late PE- (l-PE) affected pregnant women throughout pregnancy as measured by ELISA. Statistically significant difference within gestational interval is reported. Data were compared using the two-sample *t*-test between two samples or ANOVA test among more than two groups. Statistically significant difference within gestational interval is reported. SigmaPlot 12.0 and GraphPad Prism 7 were used for statistical analysis.

Similar to the analysis performed for EGFL7, levels of circulating PlGF, sFLT-1 and sENDOGLIN were measured in plasma samples from controls, isolated IUGR, and e-PE and l-PE patients along pregnancy (Fig. 2). Different from what observed for EGFL7, all three factors displayed a similar trend in isolated IUGR and in PE. Specifically, PlGF appeared significantly decreased between gestational weeks 22 and 30 in both isolated IUGR and e-PE (Fig. 2A). Between 31–34 weeks, PlGF remained significantly lower in e-PE, while levels were not significantly different in isolated IUGR and controls. No significant differences were observed at term among controls, isolated IUGR and l-PE. Considering the anti-angiogenic factor sFLT-1, both at 22–25 and at 26–30 gestational intervals its levels were significantly increased in isolated IUGR and e-PE (Fig. 2B). Although at gestational week 31–34 its levels appeared still increased compared to controls, differences were not statistically significant. At term, levels of sFLT-1 in l-PE were significantly higher compared to both isolated IUGR and controls (Fig. 2B). Similar to sFLT-1, at GA 22–25 and 26–30, sENDOGLIN was significantly increased in both isolated IUGR and e-PE (Fig. 2C). On the contrary, at 31–34 weeks, sENDOGLIN levels were significantly increased only in e-PE samples compared to both isolated IUGR and controls, which appeared to have comparable levels. At term, significantly increased levels of sENDOGLIN were measured in samples from l-PE with respect to isolated IUGR and controls (Fig. 2C).

## Discussion

In the present paper, we show that maternal circulating levels of EGFL7, a secreted protein whose function is crucial for proper placental development, are increased in PE, but not in isolated IUGR^21,29,30^. Indeed, between 22 and 25 weeks, EGFL7 levels in e-PE plasma samples were more than three times higher than those measured in controls or isolated IUGR samples (69.86 ± 6.17 vs. 19.8 ± 2.5 or 18.8 ± 2.8 µg/ml), suggesting a potential early diagnostic value of EGFL7 to discriminate between isolated IUGR and IUGR associated to PE. Such difference, although less striking, was maintained in the 26–30 weeks interval (68.69 ± 8.1 vs. 37.97 ± 9.8 µg/ml). In contrast, the know pro- and anti-angiogenic factors PlGF, sENDOGLIN and sFLT-1 showed a similar trend in IUGR and e-PE.

EGFL7 expression pattern in isolated IUGR and PE appears peculiar, since its levels are higher in e-PE compared to IUGR, and it is different from that of the other known factors PlGF, sFLT-1 and sENDOGLIN. Indeed, consistent with previous results^31^, PlGF was reduced and sFLT-1 and sENDOGLIN were increased in both types of pathological samples^32–34^. These data confirm previously published observations demonstrating that the marked increase of circulatory sFLT-1 is detectable a few weeks before the clinical manifestation of PE^35–37^. In contrast to sFLT-1, PlGF concentrations are reduced in PE, as a consequence of its binding to the increased levels of circulating sFLT-1. PlGF levels are significantly lower in women who subsequently develop PE^36,38–40^. Dysregulated expression of PlGF and sFlt-1 has been proposed to be a consequence of placental syncytiotrophoblast stress^41,42^. Similar to PE, IUGR pregnancies are characterized by elevated sFLT-1 concentrations and reduced PlGF levels^38,43–46^.

Pregnancies complicated by isolated IUGR and e-PE are both clinically affected by the most severe form of intrauterine growth compromise: the similar severity of the fetal growth disorder is revealed by the similar birthweight percentile (*p* = 0.137) although lower gestational age and lower birthweight were detected in e-PE. So that, the significant different levels of EGFL7 we observed comparing isolated IUGR and e-PE could reflect the contribution of the maternal cardiovascular system involvement and the diffuse endothelial damage to the onset of PE. Isolated IUGR is characterized by a phenotype affecting the fetus, which is not closely associated to maternal endothelial dysfunction^47^. Although PE and IUGR share a common etiopathogenesis, which is thought to be established by abnormal placentation and reduced placental perfusion^47^, in PE-affected women pro- and anti- angiogenic factors imbalance causes a maternal syndrome characterized by systemic vascular dysfunction and multiorgan damage, which is not present in women with isolated IUGR^48^. When fetal/placental alterations are associated to systemic endothelial dysfunction and multiorgan damage, as in the case of women developing PE, EGFL7 in maternal blood becomes significantly increased. Indeed, increased circulating levels of EGFL7 may be both a marker of placental dysfunction^21^, and an indicator of maternal endothelial diffuse damage. This observation is in line with our previous data in IUGR-complicated pregnancies, in which maternal cardiac adaptation was investigated by non-invasive USCOM^49^. Circulating levels of EGFL7 appeared significantly increased exclusively in IUGR patients with abnormal maternal haemodynamic profile and pathological cardiac adaptation to the pregnancy^49^.

Based on these and previous evidence, EGFL7 could be part of a panel of pro- and anti- angiogenic factors, which are known to be dysregulated in IUGR and PE^32,33^. Differently from PlGF, sFLT-1 and sENDOGLIN, its value resides in the ability to discriminate pathological conditions of pregnancy such as PE and IUGR. In fact, women with IUGR associate to e-PE are identifiable from the significant increase of EGFL7 maternal levels, while isolated IUGR, without maternal systemic involvement and diffuse endothelial damage, are characterized by low detectable maternal circulating levels of EGFL7 along all gestation.

An additional novel aspect of the reported data is the observation that increased EGFL7 levels in PE-complicated pregnancies can be revealed in the maternal blood already at the onset of the clinical manifestation (i.e., 22–25 weeks for e-PE). We had already shown that EGFL7 is altered in placenta and in blood of women with l-PE (35–40 weeks of gestation)^21,24^. Here, we show that placental levels of EGFL7 at term are similar between isolated IUGR and l-PE and are significantly lower than those measured in healthy controls. Similarly, circulating levels are not significantly different between the two groups, although levels in l-PE are significantly higher than in controls. Therefore, at term EGFL7 does not discriminate between late onset PE and IUGR, differently from what observed at the earlier gestational stages. Indeed, at 22–25 weeks of gestation, EGFL7 is significantly increased exclusively in PE, suggesting that EGFL7 could be a novel additional early marker to identify PE.

Main limitation of the study is in the small number of samples analyzed at earlier gestational age: our results should be confirmed in a larger multicenter prospective study that may be able to confirm our data also in the first and second trimester before the onset of clinical signs. A prospective study would also allow to evaluate the possible predictive value of EGFL7.

Altogether our findings indicate that circulating levels of EGFL7 are dramatically different in isolated IUGR compared to IUGR associated to e-PE already at the beginning of the second trimester of pregnancy, suggesting that EGFL7 may help for the early discrimination of the two conditions. It would be interesting to investigate if increased levels of EGFL7 can be detected before the onset of clinical manifestations and might represent the ideal angiogenetic factor to distinguish between isolated IUGR and e-PE, for which a prospective larger observational study in low and high-risk pregnancies would be needed. Indeed, EGFL7 provides additional information compared to the other known angiogenic factors, whose levels in the two conditions do not appear significantly different.
